# Relationship between Social Demographic Factors and Survival within One Year of Hospital Discharge in a Cohort of Elderly Male Patients

**DOI:** 10.2188/jea.13.203

**Published:** 2007-11-30

**Authors:** Longjian Liu, Dennis H. Sullivan

**Affiliations:** 1 Geriatric Research, Education and Clinical Center, Central Arkansas Veterans Healthcare System; Donald W. Reynolds Department of Geriatrics, University of Arkansas for Medical Sciences.

**Keywords:** social demographics, post-discharge mortality, prospective study, elderly

## Abstract

BACKGROUND: Little is known about the impact of social demographic factors on post-discharge mortality among the hospitalized elderly.

METHODS: A one-year prospective study was conducted in a random sample of 646 male patients aged 65 or older who were discharged from a university affiliated Veterans Administration Hospital at Little Rock, AR, USA. Within 48 hours of admission, each subject completed a standardized diagnostic evaluation. Mortality was recorded for all causes. Associations between patient characteristics at hospital discharge and mortality were identified utilizing univariable and multivariable (Cox proportional hazard regression) statistical techniques.

RESULTS: The mean (SD) age was 73(±6) years. Within one year of hospital discharge, 83 patients (13%) died. Multiple social demographic factors were significantly associated with mortality in univariable analysis. After controlling for age, Katz index of ADL score, Charlson co-morbidity index and length of hospitalization, risk of one-year post-discharge mortality remained significantly associated with marital status, race, education, and occupational class. When all of the social demographic factors were included in a stepwise procedure, marital status, education and occupational class were selected as the strongest predictors of mortality. The adjusted hazard ratios (95% confidence interval) of mortality associated with non-married status, education <6 years, and history of having a blue-collar occupation were 2.01 (1.29-3.15), 1.86 (1.05-3.32), and 2.16 (1.03-4.54) respectively.

CONCLUSION: The results suggest that social demographic factors are independent determinants of mortality among elderly patients. These factors should be used as important indices in identifying patients at higher risk of death in clinical assessments and in prevention programs for elderly patients after hospital discharge.

Social demographic factors may play an important role in the long-term outcomes among older patients discharged from acute care hospitals. A number of community-based studies indicate that social demographic factors such as age, race, marital status, education, occupation, income and health insurance coverage are associated with mortality.^1^^-^^12^ We also know that disease and biological indicators are important predictors of mortality among hospitalized and post-discharge elderly patients.^13^^-^^16^ However, little is known about the relationship between social demographic factors and post-discharge mortality in elderly patients after controlling for illness severity. The increasing number of elderly patients surviving serious disease conditions and the existence of social economic disparities amplify the need for studies that identify the independent impact of social demographic factors on mortality among elderly patients. Such studies could contribute to our understanding of multi-faceted determinants of patients’ survival and help in targeting clinical and preventative efforts to those in need. In order to investigate the relationship between social demographic factors and risk of mortality, we conducted a prospective study of non-terminally ill elderly patients who were being discharged from an acute care hospital. The specific objective was to determine whether social demographic factors were associated with an increased risk of death within one year of hospital discharge after controlling for illness severity and other covariates.

## METHODS

### Participants

From January 1994 through February 1997, all patients aged 65 years or older admitted to a general medical or surgical ward of the university-affiliated Department of Veterans Administration Hospital in Little Rock, Arkansas, USA, were screened within 12 hours of admission to determine eligibility. Patients with metastatic cancer and those receiving palliative care for other terminal conditions were excluded since such patients may be expected to receive less aggressive medical interventions. Remaining patients were assigned a random number. To maintain an enrollment rate of 4 to 5 patients per week, only those with a number below a pre-established cutoff were asked to enter the study. Of the 722 patients selected for the study, 44 declined to participate. Among the 678 participants (94%) who agreed to study participation, 18 died in the hospital. The remaining 660 patients were tracked for one year subsequent to discharge. Each of the 660 participants received oral and written explanations of the nature of the study and the possible risk involved prior to signing an informed consent in accordance with the ethical standards of the U.S. Department of Veterans Affairs and the Human Research Advisory Committee of the University of Arkansas for Medical Sciences. Since there were so few female cases (n=14) and gender may influence the association between social demographic factors and mortality, only male patients were included in the present data analyses. Therefore, the final study sample included 646 male patients.

### Data Collection

Within 48 hours of admission and again at discharge, each subject completed a standardized diagnostic evaluation, including social demographic factors and basic medical and functional assessments. The details of this evaluation have been published elsewhere.^15^^-^^16^ In the present analyses, five major indicators of social demographic factors were considered: (a) race (black vs. white), (b) marital status (single, married, divorced/separated and widowed), (c) occupational class (blue vs. white collar), (d) home-ownership (no vs. yes), and (e) education (years). Race was obtained from patients’ inquiry. Fewer than 1% (n=2) listed their race as other than black or white. Subjects’ occupational status was categorized as two groups (white and blue collars) based on their main lifetime occupation. White-collar group included professionals, skilled workers and clerical workers. Blue-collar group included semi-skilled and unskilled workers.^17^ Subsequent to discharge, all subjects were tracked via telephone for one year. When necessary, the VA computer system (an electronic medical record that maintained up-to-date information on patients’ home address and phone number and alternate contacts — since patients continue to receive benefits from the VA), was used to find updated phone numbers for subjects who changed residences during the year of observation.

Prior studies have indicated smoking, alcohol abuse, and illness severity indices are important correlates of mortality.^15^^,^^18^^,^^19^ We evaluated these indices as control variables (covariates) in the multivariable analyses. A patient was considered a current smoker if he smoked one or more packs of cigarettes per week within the three months prior to admission. This was confirmed by patient query and medical record review. A diagnosis of alcohol abuse was taken from the medical record and was defined as health or social problems resulting from excess alcohol consumption within the prior five years. Three indicators of illness severity were used in the study: Katz Index of Activities of Daily Living (ADL) scale,^20^ Charlson Weighted Index of Co-morbidity,^21^ and length of hospitalization (days).

### Statistical analyses

The relationship between the patient characteristics (social demographic factors and non-social demographic factors) and mortality was first examined using univariable analyses. Because of the lower educational levels in the study population, subjects’ educational status was categorized as three groups: <6 years, 6 - <9 years, and ≥9 years. In the second group of analyses, multivariable Cox proportional hazards regression models were fitted to obtain relative risk estimates of mortality. In the model, hazard ratios (analogous to relative risk) of mortality associated with study factors were calculated using Analysis of Maximum Likelihood Estimation, and the fitness of the regress model was tested using Likelihood Ratio Chi -Square Test.^22^^,^^23^ In the present study, age and three illness severity indices were controlled. The three illness severity indices were dichotomized as: Katz index of ADL score ≥2 vs. <2; Charlson co-morbidity index ≥3 vs. <3; and length of hospitalization ≥10 vs. <10 days. These cutoff points were based on their 75th percentile distributions of the total sample. Test for linear trend of hazard ratios of mortality associated with years of education (≥9, 6-<9 and <6) was also examined.^23^ In the test, the three groups of education class (i.e. ≥9, 6-<9 and <6) were coded as a new variable with values 0, 1, and 2.

In the third group of analyses, to identify the strongest social demographic predictors of mortality, all of the variables were entered into the Cox proportional hazards analysis using a stepwise procedure. A possible interaction between predictor variables was also evaluated. This was done by forcing the two predictors of interest into the Cox regression analysis along with a dummy variable set equal to the product of the two variables.^22^^,^^24^^,^^25^ Since SAS program requires creation of a new variable for the interaction term, we used the conventional method by coding the predictors (*χ*) of interest as dichotomous variables (i.e. married vs. non-married, blue vs. white collar, and education level <6 years vs. ≥6 years), and then each interaction term was created by the product of the two variables (e.g. *χ*^1^* *χ*^2^).^25^ To provide a graphic representation of the results, estimates of the survivorship function as generated by the Cox proportional hazards model for specific sets of covariate values were plotted. All data analyses were conducted using SAS^®^ software (version 8.0, SAS Institute, Cary, NC).^26^ A two-sided value of p<0.05 was considered significant.

## RESULTS

### Basic characteristics and univariable analysis

The study population had an average age of 73 ± 6 years (range, 65 to 99 years). None of the 646 participants were lost to follow-up in the year of observation. The 10 most prevalent active medical problems at admission are shown in Table 1. More than 40 % of the subjects had a problem in one or more of the following disease categories: hypertension, coronary heart disease, pulmonary disease, arthritis, or gastrointestinal disease.

**Table 1.** Most prevalent active medical problems at study entry ^a,b^

Diagnostic category^c^	%
Hypertension	55.4
CHD	47.7
Pulmonary disease	45.8
Arthritis disease	44.3
Gastrointestinal disease	42.1
Anemia	26.6
Cerebrovascular disease	25.9
Arrhythmia	25.9
Congestive heart failure	25.4
Diabetes	25.1

a: Included primary and secondary diagnosis.

b: Percent of patients with at least one of conditions from the given diagnostic category.

c: CHD: Included coronary heart disease and angina. Pulmonary disease: Included chronic obstructive pulmonary disease, bronchitis, pneumonia. Gastrointestinal disease: Included liver disease, and upper and lower gastrointestinal disorders.

Within the one-year follow-up period, 83 patients died (13%). As shown in Table 2, age, race, marital status, education, occupational class, home-ownership and indices of illness severity were significantly associated with the outcome (p<0.05, p<0.01, or p<0.001). Smoking and alcohol abuse were not significantly associated with the outcome (p>0.05). For this reason, they were not used as control variables in the multivariable analyses.

**Table 2.**
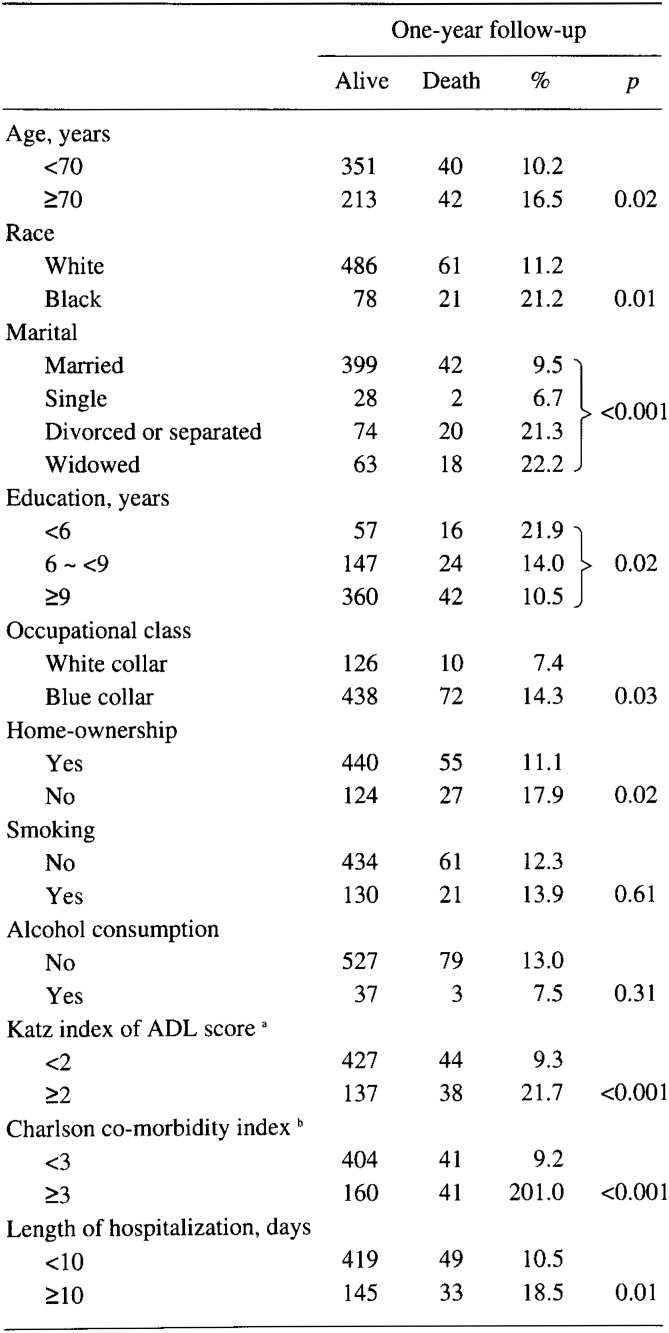
Characteristics of participants (n=646)

a: Katz index of activities of daily living (ADL) scale.

b: Charlson weighted index of co-morbidity.

### Multivariable analyses

Three models were fitted to estimate adjusted hazard ratios of mortality associated with each social demographic factor. The first (Model 1) adjusted for age, the second (Model 2) for illness severity, and the third (Model 3) adjusted for age and illness severity. Table 3 shows crude and adjusted hazard ratios (HR) of mortality and the associated 95% confidential intervals for each of the social demographic factors. Other than home-ownership, each social demographic factor remained significantly associated with mortality after controlling for age and illness severity (Models 2-3). As shown, the HR of mortality was greater for subjects who were divorced/separated (HR [95% confidence interval]: 1.50 [1.15-1.96]) and widowed (1.35[1.12-1.62]) compared to married subjects (Model 3). The HRs (95% confidence interval) of mortality in subjects who received 6 - <9 years of education and those who received <6 years of education were 1.28 (0.78-2.11) and 2.34 (1.30-4.19) respectively, (test for linear trend, p<0.01) compared to those with education ≥9 years.

**Table 3.** Hazard ratios for one-year post-discharge mortality by five social demographic factors ^a^

	No.	crude hazard ratio		Adjusted hazard ratio					

				Model 1		Model 2		Model 3	
Marital status:									
Non-married (vs. married) ^b^	205(441)	2.23	(1.45 - 3.44)	2.21	(1.44 - 3.41)	2.13	(1.38 - 3.27)	2.10	(1.37 - 3.24)
Marital status by four groups									
Married	441	1		1					
Single	30	1.01	(0.31 - 3.27)	1.00	(0.31 - 3.23)	0.95	(0.29 - 3.06)	0.93	(0.29 - 3.00)
Divorced or separated	94	1.54	(1.18 - 2.01)	1.56	(1.19 - 2.03)	1.50	(1.15 - 1.96)	1.50	(1.15 - 1.96)
Widowed	81	1.37	(1.14 - 1.65)	1.36	(1.13 - 1.63)	1.36	(1.13 - 1.63)	1.35	(1.12 - 1.62)
Education level, years									
Lower education (vs. higher) ^c^	73(573)	2.02	(1.17 - 3.48)	1.97	(1.14 - 3.41)	2.20	(1.26 - 3.82)	2.15	(1.23 - 3.74)
Education by 3 groups									
≥9	402	1		1					
6 - <9	171	1.44	(0.88 - 2.36)	1.39	(0.84 - 2.28)	1.30	(0.39 - 2.14)	1.28	(0.78 - 2.11)
<6	73	2.27	(1.28 - 4.04)	2.20	(1.24 - 3.92)	2.40	(1.34 - 4.30)	2.34	(1.30 - 4.19)
*Test for linear trend ^d^*	646	*p=0.0048*		*P=0.0075*		*p=0.0057*		*p=0.0077*	

Race: Black (vs. white)	99(547)	1.95	(1.19 - 3.20)	1.91	(1.17 - 3.14)	1.85	(1.12 - 3.04)	1.81	(1.10 - 2.98)
Occupation: Blue collar (vs. white collar)	510(136)	2.01	(1.04 - 3.89)	1.97	(1.02 - 3.82)	2.11	(1.09 - 4.09)	2.08	(1.08 - 4.04)
Home-ownership: No (vs. yes)	151(495)	1.75	(1.10 - 2.78)	1.72	(1.08 - 2.74)	1.52	(0.95 - 2.42)	1.53	(0.96 - 2.44)

95% confidence intervals in parentheses.

a: Model 1: adjustment for age. Model 2: adjusted for Katz index of ADL score, Charlson co-morbidity index and length of hospitalization. Model 3: adjustment for all in models 1 and 2.

b: Non-married included single, divorced/separated and widowed.

c: Lower education: < 6 years, and higher education: ≥6 years.

d: Test for linear trend: Education level was coded as 0, 1, 2 for those with education ≥9, 6-<9 and < 6 years respectively.

After forcing the control variables (used in Table 3, Model 3) into the Cox regression model, a stepwise procedure was utilized to select the strongest social demographic predictors. In this analyses, to summarize the relative risks for subjects with unmarried status and those with educational level < 6 years, these two variables were grouped as unmarried (including single, divorced/separated and widowed) vs. married; and educational level <6 years vs. ≥6 years. The cut-off point for education was taken as the completion of primary school (six years). Table 4 shows that of the five social demographic variables that were included in the analysis, only marital status, followed by education and occupational class entered the final model (likelihood ratio *χ*^2^ = 57.9 with 7 df, p<0.001). This final Cox regression model was retained.

**Table 4.** The relationship between one-year post-discharge mortality and each social demographic factor that entered the final model when the stepwise procedure was utilized ^a^

	Covariates	Adjusted Hazard Ratio (95% confidence interval)	
Steps			
1	Marital status (non-married vs. married)	2.01	(1.29 - 3.15)
2	Education (< 6 vs. ≥ 6 years)	1.86	(1.05 - 3.32)
3	Occupational class (blue collar vs. white collar)	2.16	(1.03 - 4.54)
Force entry			
	Age (≥ 75 vs. < 75 years)	1.21	(0.77 - 1.91)
	Katz index of ADL score (≥ 2 vs. < 2)	2.14	(1.34 - 3.42)
	Charlson co-morbidity index (≥ 3 vs. < 3)	2.20	(1.40 - 3.46)
	Length of hospitalization (≥ 10 vs. < 10 days)	1.51	(0.94 - 2.42)
*Likelihood ratio χ^2^ = 57.9, df=7, P<0.001*			

a: All five social demographic factors (marital status, education, race, occupational class and home-ownership) were entered into a stepwise procedure after age, Katz index of ADL score, Charlson’s co-morbidity index and length of hospitalization were forced into the model. As shown, marital status, education, and occupational class entered into the final model at p<0.05.

**Figure 1.**
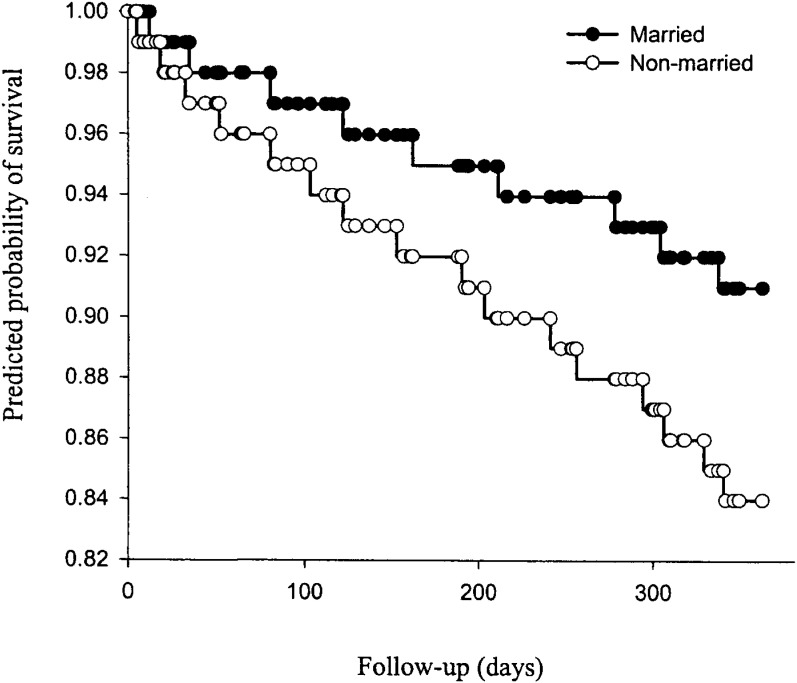
Adjusted survival probability by marital status plotted as a function of days since hospital discharge. The survivorship estimates used to produce these survival curves were generated by the Cox proportional hazards model for each value of marital status (0 or 1) while controlling for age and illness severity.

Using the retained model, the estimated survivorship function for a hypothetical married patient was contrasted with that of another patient who differed only in that he was not married. These results are depicted graphically in Figure 1. By controlling for the other six variables in the model, the independent effect of marital status on mortality is demonstrated. There is a similar relationship between education on mortality and occupational class on mortality (data not shown). Tests for interaction between marital status and education (HR [95% confidence interval]: 0.86 [0.23 - 2.58], p=0.79), marital status and occupational class (0.89 [0.23 - 3.40], p=0.86), and education and occupational class (0.14 [0.02 - 1.16], p=0.07) were not significant.

## DISCUSSION

Although it is known that patients’ disease status and other biological indicators may be strongly associated with post-discharge mortality, the present study provides further evidence that certain social demographic factors are also important determinants of mortality among elderly patients subsequent to hospital discharge. Compared to those who were married, older non-married patients were more than twice as likely to die within the year subsequent to discharge. There was a similar level of risk associated with having had less than 6 years of education and having had a blue-collar occupation. Even after controlling for age and illness severity, the relationship between these social demographic factors and the outcome remained significant (Table 4). This finding highlights the important independent association between these social demographic factors and mortality in the study population.

Although an association between social demographic factors and mortality has been demonstrated in prior studies of older individuals,^6^^-^^12^ their importance as predictors of post-hospital discharge mortality remained to be defined. It was possible that other potentially more powerful determinants of survival in the year subsequent to hospital discharge offset any possible effects of the social demographic factors on this outcome. Since age and illness severity have been shown to be powerful predictors of post-discharge mortality among older patients,^6^^,^^14^ we controlled for these variables in the analyses. When we examined marital status, years of education, and occupational class, the results indicated that age and illness severity did not change the relationship between these social demographic factors and mortality.

The relationship between marital status and mortality has been examined previously in several different populations of hospitalized patients.^2^^,^^4^^,^^11^^,^^27^ Gordon and Rosenthal et al. examined 40,820 adult patients and found the relative risk of in-hospital death among non-married surgical patients was more than 1.3 times greater (95% confidence interval: 1.06-1.58) than among married surgical patients.^27^ In a study of the impact of marital status on cancer survival among Norwegian population, never-married men, never-married women and divorced men had an overall mortality elevated by about 15% as compared with married men and women.^11^ In the present study, we found a similar relationship between marital status and mortality among a cohort of elderly male patients during the year following hospital discharge. Even after adjusting for age and illness severity, the relative risk of death for those who were not married was more than two times that of the remaining subjects. When all of the social demographic factors were included in a stepwise procedure, marital status was the first factor to be selected for entry into the Cox prediction model that already included age and the illness severity variables. The association between marital status and mortality remained significant after both education and occupational class entered the model. These results suggest that marital status may be an important determinant of survival in select older populations. There are several possibilities as to why this may be true. One explanation is that marital status may function as a measure of social support. For example, married patients may be more likely to have a caregiving spouse or financial resources that are adequate to ensure that timely and effective medical care is provided when needed. Spousal support may also modify or alleviate emotional stress that may produce physiologic benefit by unknown neural and/or neurohumoral pathways.^27^ Further studies are needed to explore the mechanisms.

Although marital status is beyond the control of healthcare professionals, the results of the present study emphasize the potential advantage of using social demographic factors in risk assessment to identify individuals who may benefit from interventions that bolster their social support networks.

Education and occupational class tire most commonly used as indictors of social economic status. The impact of social economic disparities on mortality has been studied in several populations and countries. The results are mostly consistent, the lower the social economic status the higher the mortality rate.^3^^,^^6^^,^^7^^,^^9^ The Finnish Cancer Registry study investigated the association between social class (defined by using occupational status) and survivals in 106,661 cancer patients aged 25 to 79 years. It showed that age-adjusted relative risk of death (after 5 years follow-up) due to cancer was highest for those in the lowest social class in both men and women.^7^ This is consistent with our findings. Education and occupational class were both significantly associated with one-year post-discharge mortality. This association may be attributable to the relationship of social economic status to healthy behaviors and the use of medical care service. Home-ownership has also been evaluated as an indicator of social economic status and found to be an important predictor of health outcomes in several studies.^3^^,^^28^^,^^29^ However, in our study, homeownership had a non-significant relationship with mortality after adjustment for illness severity. This finding suggests that this variable may provide little additional explanatory power of social economic status on outcomes.

Consistent with other studies,^5^^,^^10^ we found black patients had a higher adjusted relative risk of mortality compared to white patients in both the univariable and multivariable analyses (Tables 2 and 3). However, when we controlled for other social demographic factors in the stepwise model (Table 4), race no longer was significant implying there may be differences in education or income that explain the higher risk for blacks than whites.

It should be noted that several limitations might exist in our present study. First, the findings may have limitation in its generalizability, since the participants were recruited from a veteran affairs hospital. However, since there are more than 171 veteran medical centers/hospitals and more than 119 nursing home care facilities across the USA, which serve for more than 24 million veterans (more than 8.3 million of them are at age 65 or older), and similar healthcare systems in other countries,^30^ the present findings may add to the knowledge in the study populations. Second, the association between demographic factors and mortality data from a specific disease was not tested. In the study, we collected the mortality from all causes of death, partly because we believe that it may be less important to test the disease-specific mortality among the older patients due to their multiple morbidity status. Third, the subjects’ smoking status was classified into two groups - current smokers and non-smokers. The non-smoking group included subjects who had smoked before but quit smoking at the study entry (i.e. ex-smokers). We had no exact data on the ex-smoking status. The long-term hazards of smoking on health therefore may be underestimated in the present study. Fourth, the question of alcohol abuse is a somewhat sensitive one. In addition to the accuracy of data obtained, the sample size of the present study might be small and unable to test the alcohol abuse — mortality association. Further studies are certainly needed.

Nevertheless, the findings of the present study suggest that social demographic factors have an independent impact on post-discharge mortality among elderly patients. This implies that social support and social economic status may play a very important role in improving the health outcome. For this reason, social demographic factors should be used as important indices in identifying patients at higher risk of death in clinical assessments and in prevention programs for the elderly patients. In this study, subjects were followed for one year subsequent to hospital discharge. It remains to be determined whether the associations between the social demographic factors and mortality remain significant after this time period.
